# The slope associated with nadir prostate‐specific antigen is prognostically significant in men with hormone‐sensitive prostate cancer after primary androgen deprivation therapy

**DOI:** 10.1002/cam4.4685

**Published:** 2022-03-21

**Authors:** Zeng Zhenhao, Cheng Xiaofeng, Jiang Hao, Yi Ming, Zhang Hongtao, He Wenrui, Zhang Cheng, Zhou Xiaochen, Wang Gongxian

**Affiliations:** ^1^ Department of Urology The First Affiliated Hospital of Nanchang University Nanchang China; ^2^ Jiangxi Institute of Urology Nanchang China; ^3^ Department of Ultrasound Medicine The Second Affiliated Hospital of Nanchang University Nanchang China

**Keywords:** androgen deprivation therapy, hormone‐sensitive prostate cancer, nadir prostate‐specific antigen, nadir prostate‐specific antigen declining slope, slope associated with nadir PSA

## Abstract

**Background:**

Prognostic indicators based on the initial prostate‐specific antigen (PSA) levels, nadir PSA, and time to PSA nadir were calculated to evaluate prognosis after primary androgen deprivation therapy (PADT), as these have been reported in very few studies. We attempted to evaluate the prognostic role of the slope associated with nadir PSA in patients treated with PADT.

**Methods:**

A total of 107 patients who were treated with PADT from 2015 to 2019 were reviewed. The Kaplan–Meier method and Cox regression model were used to analyze the prognostic significance of the slope associated with nadir PSA in predicting progression‐free survival (PFS) and overall survival (OS).

**Results:**

After PADT, the median follow‐up duration was 40.1 months; 66 patients (61.7%) had disease progression, and 33 patients (30.8%) died. In the univariate analysis, T stage, N stage, nadir PSA, time to PSA nadir, nadir PSA declining slope (nPSA‐DS), nadir PSA percentage declining slope (nPSA‐PDS), and nadir PSA line slope (nPSA‐LS) were significant predictors for PFS and OS. The multivariate analysis showed that a higher nPSA‐DS (> − 0.74) and lower PSA nadir (≤0.16 ng/ml) were independent predictors for prolonged survival. The significance of nPSA‐DS and nPSA was supported by the analysis of nPSA‐DS and nPSA as time‐dependent covariates. The combined analyses demonstrated that patients with a higher nPSA‐DS and lower PSA nadir had the best PFS and OS.

**Conclusions:**

The slope associated with the nadir PSA of nPSA‐DS was a significant independent predictor for patients treated with PADT. Nadir PSA and nPSA‐DS have a synergistic effect on prognosis.

## 1 INTRODUCTION

Prostate cancer (PCa) is the second most common malignant tumor in men and the fifth leading cause of death in cancer patients,^1^ with an estimated nearly 375,000 deaths and 1.4 million new cases of prostate cancer worldwide in 2020.^1^ Androgen deprivation therapy (ADT) plays essential roles in advanced hormone‐sensitive prostate cancer (HSPC) patients.^2^, ^3^ In Asia, ADT is still an essential part of treating localized and metastatic PCa^4^ and is recommended even in the case of relatively early‐stage diagnosis of PCa.^5^ However, many patients will eventually develop castration‐resistant prostate cancer (CRPC), which is associated with a poor prognosis and a high rate of metastasis.

Prostate‐specific antigen (PSA) is not only widely used for PCa screening, but also as a useful prognostic indicator in various clinical settings.^6^, ^7^, ^8^ PSA values at the initial diagnosis correlate with prognosis. In general, the higher the level of PSA, the worse the outcome will be.^9^ The J‐CAPRA score is an effective indicator of prognosis in patients receiving primary androgen deprivation therapy (PADT), and initial PSA (iPSA) is a crucial component of the J‐CAPRA score.^10^, ^11^ Nadir PSA (nPSA) and time to PSA nadir (TTPN) have been demonstrated to be useful indicators for predicting disease progression or survival in patients with HSPC.^12^, ^13^, ^14^

A declining pattern of PSA, nadir PSA, TTPN, and PSA percent decline after treatment with ADT has been reported to be associated with prognosis.^14^, ^15^, ^16^ However, the roles of the slope associated with nadir PSA based on initial PSA, PSA nadir, and TTPN in evaluating the prognosis of prostate cancer patients after treatment have been reported in very few studies. Therefore, we obtained the slope associated with nadir PSA according to different calculation methods.^12^, ^15^, ^17^ These methods included the nPSA declining slope (nPSA‐DS), nPSA percentage declining slope (nPSA‐PDS), and nPSA line slope (nPSA‐LS), which were defined as the slope associated with nadir PSA from a mathematical point of view according to the calculation formula. Then, we investigated whether the slope associated with nadir PSA could be utilized as a prognostic indicator in men with prostate cancer undergoing ADT. We believe that the slope associated with nadir PSA can be helpful for clinical treatment strategies and predicting the treatment response.

## 2 MATERIALS AND METHODS

From January 2015 to June 2019, we retrospectively collected detailed clinical data on men with histologically confirmed prostate cancer in our hospital. The total number of HSPC patients who received PADT were 107. PADT was defined as taking luteinizing hormone‐releasing hormone (LHRH) agonists or bilateral orchiectomy combined with antiandrogens such as bicalutamide. The patient inclusion criteria were one or more PSA values available less than 1 month before the start of PADT and ≥2 PSA values available before nPSA (including nPSA).^12^ If patients' PSA levels increased after the start of PADT or only two PSA values were available and the second PSA was obtained more than 1 year after the first record, they were excluded from the analysis.^12^ The exclusion criteria were other primary/secondary malignancies; metabolic and systemic diseases that seriously affect treatment and life expectancy (heart failure, acute cerebral infarction, respiratory failure, etc.), and previous treatment, such as radiation therapy or prostatectomy.

The clinical, biological, and pathological characteristics of patients were obtained from medical records and included age, iPSA, clinical TNM stage, Gleason score, nPSA level, TTPN, and mode of PADT. nPSA was defined as the minimal PSA value first observed after PADT. TTPN was defined as the duration needed for the nPSA value to first be observed after PADT initiation. nPSA‐DS, nPSA‐PDS, and nPSA‐LS were defined as [nPSA‐DS = (ln (iPSA)−ln (nPSA))/TTPN], {nPSA‐PDS = [100* (iPSA‐nPSA/iPSA)]/TTPN}, and [nPSA‐LS = (iPSA‐nPSA)/TTPN], respectively.

Progression‐free survival (PFS) and overall survival (OS) were the primary study endpoints. Progression was defined as two increases in PSA (>7 days apart) greater than the increase in nPSA.^18^ The start of any second‐line therapy for increased PSA was also regarded as a progression event.^18^ The time from the initiation of PADT to death was defined as OS.

Continuous variables with a normal distribution are expressed as the mean (SD), and other continuous variables are expressed as the median (IQR). Categorical variables are summarized as the number (%). The median was used as a cutoff value for continuous variable grouping. Continuous variables, including age, iPSA, nPSA, TTPN, nPSA‐DS, nPSA‐PDS, and nPSA‐LS, were analyzed using independent *t* tests or the Wilcoxon‐rank sum test.^19^ The categorical variables included the clinical TNM stage, Gleason score, and mode of PADT. Pearson's χ^2^ test or Fisher's test^20^ was used to analyze categorical variables. Multivariate Cox hazard proportion (forward stepwise likelihood ratio mode)^21^ analyses were performed to confirm the significant factor for predicted PFS and OS. We also performed Cox proportional hazards regression^21^ analysis with nPSA, TTPN, nPSA‐DS, nPSA‐PDS, and nPSA‐LS as time‐dependent covariates to reduce the effect of immortal time bias. Kaplan–Meier analysis^22^, ^23^ was performed to estimate PFS and OS. Significance was assessed by using the log‐rank test.^22^, ^23^ *p* < 0.05 was considered statistically significant. All statistical analyses were performed using GraphPad/Prism 8.0 and SPSS 22.0 software.

## 3 RESULTS

The clinicopathological characteristics of 107 prostate cancer patients who underwent PADT are listed in Table 1. The mean age and median age of the cohort of patients at PADT initiation were 71.74 and 72 years, respectively. The median follow‐up time was 40.07 months, and the treatment of PADT was LHRH agonists combined with antiandrogen was given in 88.8% of the patients. Sixty‐six patients (61.7%) had disease progression, and 33 patients (30.8%) died. Most of the patients had clinical metastatic disease (N1, 42.1%; M1, 70.1%) at diagnosis, and 72.9% of the cohort had a Gleason score of 8 to 10. The median iPSA was 113.5 ng/ml. The median nPSA and TTPN were 0.16 ng/ml and 7.93 months, respectively.

**TABLE 1:** clinical, biological, and pathological characteristics of patients

	Mean (SD)	Median (IQR)	Number (%)
At PADT initial			
Age (years)	71.74 (8.2)		
Initial PSA (ng/ml)		113.5 (43.6356.9)	
Clinical T‐stage			
T1–T2			62 (57.9)
T3–T4			45 (42.1)
Clinical N‐stage			
N0			62 (57.9)
N1			45 (42.1)
Clinical M‐stage			
M0			32 (29.9)
M1			75 (70.1)
Gleason score			
≤7			29 (27.1)
8–10			78 (72.9)
Mode of PADT			
LHRH agonists+antiandrogen			95 (88.8)
Bil.orchiectomy+antiandrogen			12 (11.2)
During PADT			
Nadir PSA (ng/ml)		0.16 (0.02,1.08)	
TTPN (months)		7.93 (3.97,15.37)	
Slope associated with nadir PSA			
nPSA‐DS		−0.74 (−1.28,−0.39)	
nPSA‐PDS		12.52 (6.51,25.17)	
nPSA‐LS		−14.38 (−56.52,−3.93)	

Abbreviations: Bil.orchiectomy, bilateral orchiectomy; IQR, interquartile range; LHRH, luteinizing hormone‐releasing hormone; nPSA‐DS, nadir PSA declining slope; nPSA‐LS, nadir PSA line slope.; nPSA‐PDS, nadir PSA percentage declining slope; PADT, primary androgen deprivation therapy; SD, standard deviation; TTPN, time to PSA nadir.

**FIGURE 1:**
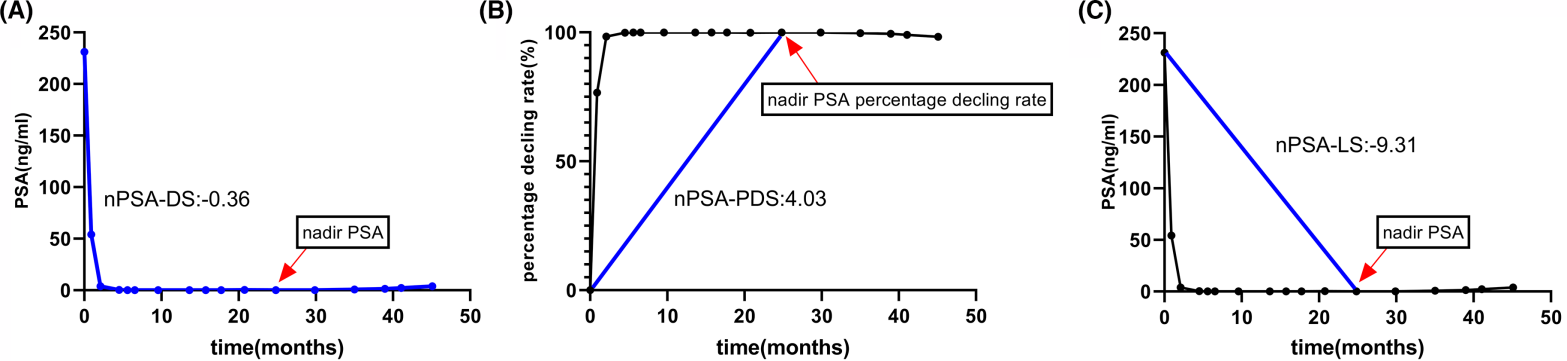
Calculation of the slope associated with nadir PSA for a sample patient after primary androgen deprivation therapy. (A) Nadir PSA declining slope (nPSA‐DS), (B) nadir PSA percentage declining slope (nPSA‐PDS), (C) nadir PSA line slope (nPSA‐LS)

In order to investigate the slope associated with nadir PSA in evaluating prognosis after PADT. The slope associated with nadir PSA was calculated based on initial PSA, nadir PSA, and TTPN. Figure 1 is an illustrative example from the same patient treated with PADT. The median values of the cohort of patients were −0.74, 12.52 and −14.38 for different slopes of nPSA‐DS, nPSA‐PDS, and nPSA‐LS post‐PADT, respectively.

The univariate analysis of PFS showed that age, initial PSA, clinical TNM stage, Gleason score, nadir PSA, TTPN, nPSA‐DS, nPSA‐PDS, and nPSA‐LS affected PFS (Table 2). The multivariate analysis revealed that age ≥ 72 years (HR, 0.58; 95% CI 0.34–0.99; *p* = 0.044), N1 group (HR, 2.68; 95% CI 1.56–4.61; *p* < 0.001), nadir PSA >0.16 ng/ml (HR, 17.99; 95% CI 8.88–36.49; *p* < 0.001), and nPSA‐DS > −0.74 (HR, 0.30; 95% CI 0.18–0.52; *p* < 0.001) were significant independent prognostic factors of PFS (Table 2).

**TABLE 2:** Univariate and multivariate Cox regression analyses of the clinicopathologic factors predict progression‐free survival

Factors	Univariate		Multivariate	
	HR (95% CI)	*p* value	HR (95% CI)	*p* value
Age (<72 vs. ≥72)	0.46 (0.28–0.75)	0.002	0.58 (0.34–0.99)	0.044
Initial PSA (<113.5 vs. ≥113.5)	2.06 (1.25–3.40)	0.005		0.148
T stage (<T2 vs. ≥T3)	2.05 (1.26–3.34)	0.004		0.525
N stage (N0 vs N1)	2.86 (1.74–4.70)	<0.001	2.68 (1.56–4.61)	<0.001
M stage (M0 vs. M1)	3.05 (1.55–5.99)	0.001		0.148
Gleason score (<8 vs. ≥8)	2.07 (1.10–3.89)	0.024		0.055
Mode of PADT (drug vs. surgery)	0.84 (0.60–1.18)	0.312		
Nadir PSA (≤0.16 vs. >0.16)	10.95 (5.80–20.67)	<0.001	17.99 (8.88–36.49)	<0.001
TTPN (≤7.93 vs. >7.93)	0.23 (0.14–0.39)	<0.001		0.115
nPSA‐DS (≤ − 0.74 vs. >−0.74)	0.41 (0.25–0.68)	0.001	0.30 (0.18–0.52)	<0.001
nPSA‐PDS (≤12.52 vs. >12.52)	3.96 (2.37–6.61)	<0.001		0.176
nPSA‐LS (≤ − 14.38 vs. >−14.38)	0.19 (0.11–0.34)	<0.001		0.779

Abbreviations: HR, Hazard ratio; CI, confidence interval; drug, luteinizing hormone‐releasing hormone agonists+antiandrogen; nPSA‐DS, nadir PSA declining slope; nPSA‐LS, nadir PSA line slope.; nPSA‐PDS, nadir PSA percentage declining slope; PADT, primary androgen deprivation therapy; surgery, bilateral orchiectomy+antiandrogen; TTPN, time to PSA nadir;

Table 3 shows the association between each factor and overall survival. The univariate analysis demonstrated that clinical ≥T3 stage, N1, nadir PSA > 0.16 ng/ml, TTPN ≤7.93 months, nPSA‐DS ≤ −0.74, nPSA‐PDS > 12.52, and nPSA‐LS ≤−14.38 were significant risk factors for OS (*p* < 0.05). The multivariate analysis indicated that only nadir PSA > 0.16 ng/ml (HR, 7.72; 95% CI 3.15–18.96; *p* < 0.001) and nPSA‐DS > −0.74 (HR, 0.23; 95% CI 0.10–0.51; *p* < 0.001) were significant predictors associated with OS. We also performed Cox proportional hazards regression model analysis with nPSA, TTPN, nPSA‐DS, nPSA‐PDS, and nPSA‐LS as time‐dependent covariates. The multivariate Cox analysis showed that nPSA‐DS and nPSA, as time‐dependent covariates, were significantly correlated with PFS and OS (Table 5).

**TABLE 3:** Univariate and multivariate Cox regression analyses of the clinicopathologic factors predict overall survival

Factors	Univariate		Multivariate	
	HR (95% CI)	*p* value	HR (95% CI)	*p* value
Age (<72 vs. ≥72)	0.70 (0.35–1.39)	0.309		
Initial PSA (<113.5 vs. ≥113.5)	1.90 (0.95–3.83)	0.072		
T stage (<T2 vs. ≥T3)	2.4 (1.20–4.81)	0.014		0.197
N stage (N0 vs. N1)	2.87 (1.43–5.77)	0.003		0.076
M stage (M0 vs. M1)	1.43 (0.64–3.16)	0.383		
Gleason score (<8 vs. ≥8)	1.75 (0.78–3.93)	0.177		
Mode of PADT (drug vs. surgery)	0.70 (0.27–1.83)	0.469		
Nadir PSA (≤0.16 vs. >0.16)	7.20 (2.94–17.63)	<0.001	7.72 (3.15–18.96)	<0.001
TTPN (≤7.93 vs. >7.93)	0.2 (0.09–0.44)	<0.001		0.207
nPSA‐DS (≤ − 0.74 vs. >‐0.74)	0.25 (0.11–0.55)	0.001	0.23 (0.10–0.51)	<0.001
nPSA‐PDS (≤12.52 vs. >12.52)	5.2 (2.38–11.38)	<0.001		0.159
nPSA‐LS (≤ − 14.38 vs. >‐14.38)	0.29 (0.14–0.61)	0.001		0.265

Abbreviations: HR, Hazard ratio; CI, confidence interval; drug, luteinizing hormone‐releasing hormone agonists+antiandrogen; nPSA‐DS, nadir PSA declining slope; nPSA‐LS, nadir PSA line slope.; nPSA‐PDS, nadir PSA percentage declining slope; PADT, primary androgen deprivation therapy; surgery, bilateral orchiectomy+antiandrogen; TTPN, time to PSA nadir.

**FIGURE 2:**
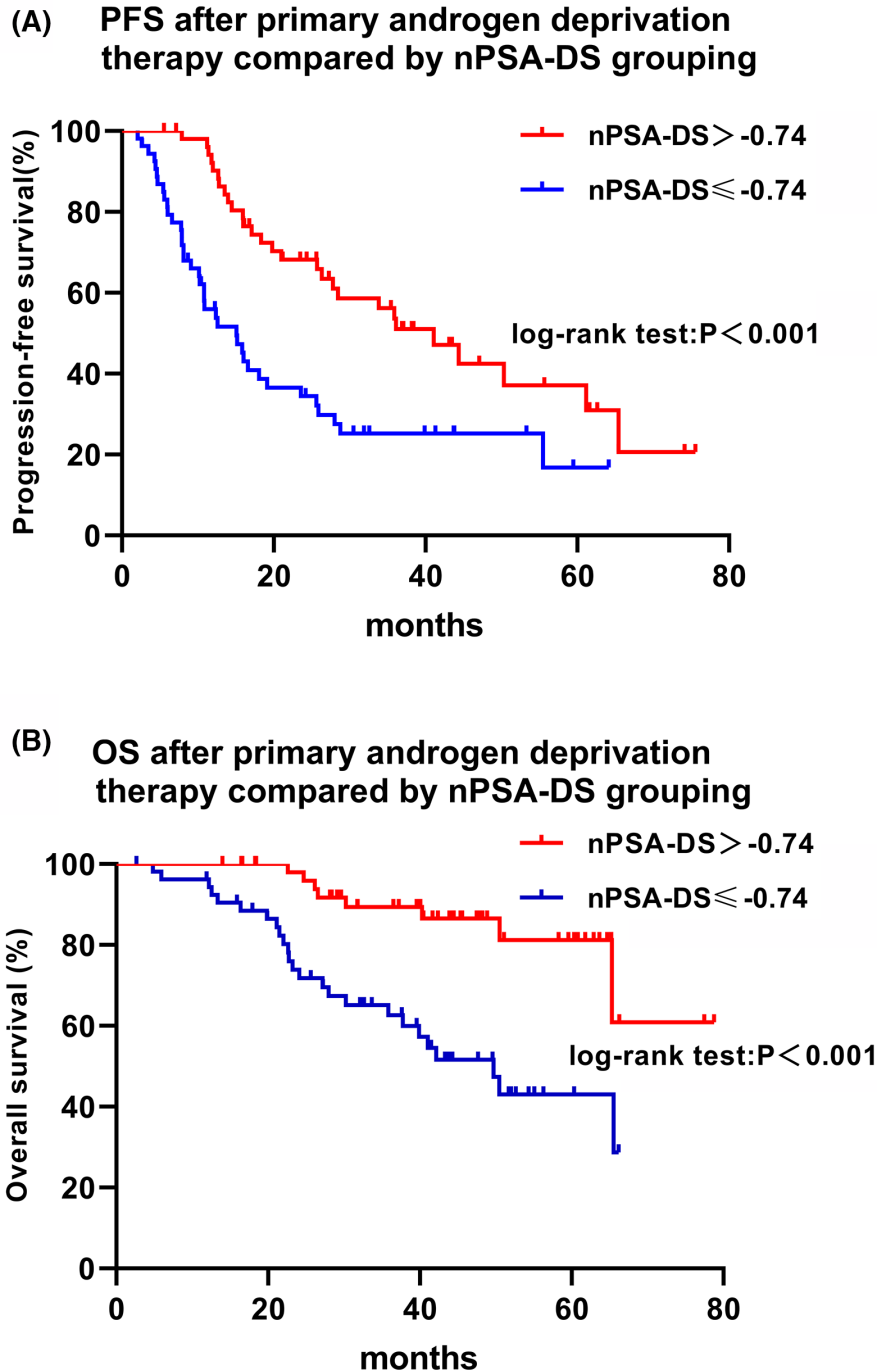
Kaplan–Meier curve estimates of the time to PFS and OS for groups classified by the cutoff value of nPSA‐DS. (A) Progression‐free survival of patients in the different nPSA‐DS groups. (B) Overall survival of patients in the different nPSA‐DS groups

We performed Kaplan–Meier survival analysis to estimate the median time of PFS and OS. Figure 2 shows PFS and OS stratified by nPSA‐DS. The median PFS duration was 41.07 months in patients with nPSA‐DS > −0.74 and 15.10 months in those with nPSA‐DS ≤ −0.74 (*p* < 0.001). The median overall survival was not attained in the case of nPSA‐DS > −0.74 versus 49.73 months in the case of nPSA‐DS ≤ −0.74 (*p* < 0.001).

**FIGURE 3:**
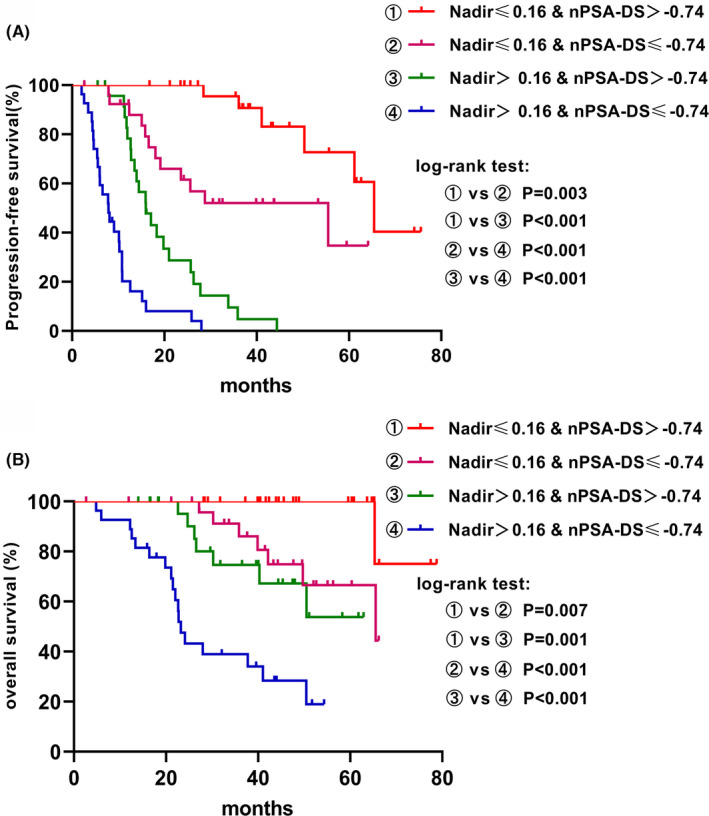
Kaplan–Meier curve estimates of the time to PFS and OS. (A) Progression‐free survival based on nadir PSA and the nPSA declining slope; (B) Overall survival based on nadir PSA and the nPSA declining slope

nPSA‐DS (≤−0.74 vs. >−0.74) after PADT showed a significant correlation with clinical N stage (*p* = 0.014), age (*p* = 0.001), and TTPN (*p* < 0.001). Initial PSA, T stage, M stage, Gleason score, and nadir PSA were not significantly different between the two groups (*p* > 0.05) (Table 4). Then, a Kaplan–Meier plot of PFS and OS by nadir PSA and nPSA‐DS indicated that lower nPSA (≤0.16 ng/ml) and a higher nPSA‐DS (> −0.74) were associated with the best PFS and OS. Conversely, higher nPSA (>0.16 ng/mL) and a lower nPSA‐DS (≤ − 0.74) were associated with the worst PFS and OS (Figure 3).

**TABLE 4:** Comparison of several factors for prostate cancer patient with PADT between nPSA‐DS ≤ −0.74 and nPSA‐DS > −0.74

Factors	nPSA‐DS ≤ −0.74 (*n* = 54)	nPSA‐D > −0.74 (*n* = 53)	*p* value
Age			
<72	34	17	0.001
≥72	20	36	
Initial PSA			
<113.5	22	31	0.066
≥113.5	32	22	
T stage			
T1–T2	27	35	0.093
T3–T4	27	18	
N stage			
N0	25	37	0.014
N1	29	16	
M stage			
M0	13	19	0.183
M1	41	34	
Gleason score			
≤7	14	15	0.782
8–10	40	38	
Nadir PSA			
≤0.16	27	28	0.770
>0.16	27	25	
TTPN			
≤7.93	44	11	<0.001
>7.93	10	42	

Abbreviations: nPSA‐DS, nadir PSA declining slope; PADT, primary androgen deprivation therapy; TTPN, time to PSA nadir.

**TABLE 5:** Multivariate Cox regression analyses with nPSA, TTPN, nPSA‐DS, nPSA‐PDS, and nPSA‐LS as time‐dependent covariates predict PFS and OS

Factors	PFS		OS	
	HR (95% CI)	*p* value	HR (95% CI)	*p* value
Age (<72 vs. ≥72)	0.52 (0.30–0.89)	0.016		
Initial PSA (<113.5 vs. ≥113.5)		0.244		
T stage (<T2 vs. ≥T3)		0.502		0.152
N stage (N0 vs. N1)	2.93 (1.70–5.04)	<0.001		0.057
M stage (M0 vs. M1)		0.179		
Gleason score (<8 vs. ≥8)		0.053		
Nadir PSA (≤0.16 vs. >0.16)	2.79 (2.19–3.56)	<0.001	1.79 (1.38–2.33)	<0.001
TTPN (≤7.93 vs. >7.93)		0.297		0.223
nPSA‐DS (≤ − 0.74 vs. >‐0.74)	0.41 (0.25–0.68)	0.001	0.66 (0.52–0.84)	0.001
nPSA‐PDS (≤12.52 vs. >12.52)		0.404		0.174
nPSA‐LS (≤ − 14.38 vs. >‐14.38)		0.974		0.242

Abbreviations: CI, confidence interval; HR, Hazard ratio; nPSA‐DS, nadir PSA declining slope; nPSA‐LS, nadir PSA line slope.; nPSA‐PDS, nadir PSA percentage declining slope; OS, overall survival; PFS, progression‐free survival; TTPN, time to PSA nadir.

## 4 DISCUSSION

A number of studies have shown TTPN and nadir PSA to be important predictors of progression and survival in prostate cancer patients after ADT.^12^, ^24^, ^25^, ^26^, ^27^, ^28^ These studies have shown that longer TTPN after PADT was significantly related to prolonged progression‐free,^25^, ^26^, ^27^, ^28^ cancer‐specific,^26^ and overall^12^, ^26^, ^28^ survival. Lower nadir PSA after PADT was a significant prognostic factor of prolonged progression‐free,^25^, ^26^, ^27^, ^28^ cancer‐specific,^25^, ^27^ and overall^12^, ^24^, ^27^, ^28^ survival. The initial PSA value has also been reported to predict prognosis after PADT in several reports.^10^, ^29^

It may be a reliable and accurate prognostic indicator that was calculated based on nadir PSA, TTPN, and initial PSA for evaluating the prognosis after PADT, but very little has been reported. Choueiri et al.^12^ calculated the PSA decline (PSAD) by linear regression of the PSA value with time and found that the patients had prolonged overall survival when PSAD was <52 ng/ml/year in the univariate analysis. Akbay et al.^15^ used a formula for assessing the PSA decline pattern to calculate the declining slope to nadir PSA (ds‐nPSA), and they found that patients with ds‐nPSA> − 0.507 had prolonged PFS in the univariate analysis. Lin et al.^17^ calculated the PSA reduction rate (PSARR) according to the formula PSARR = (100* (iPSA‐nPSA/iPSA)/TTPN). In the univariate analysis, the PSARR was a statistically significant indicator for disease progression and OS, but no statistical significance was found in the multivariate analysis.

In the present study, we aimed to define ds‐nPSA, PSAD, and the PSARR as the slope associated with the nadir PSA of nPSA‐DS, nPSA‐LS, and nPSA‐PDS, respectively, from a mathematical point of view (Figure 1). We investigated the prognostic significance of the slope associated with nadir PSA in patients treated with PADT. In accordance with previous reports,^12^, ^15^, ^17^ our results also showed that nPSA‐DS, nPSA‐LS, and nPSA‐PDS were associated with PFS and OS in the univariate analysis. Moreover, we found that nadir PSA and nPSA‐DS following PADT were independently associated with PFS and OS, even after adjusting for other factors. Nadir PSA (≤0.16 ng/ml) and the slope of nPSA‐DS (> − 0.74) were independent predictors of prolonged PFS and OS in patients with PADT. The estimated median PFS and OS times in the nPSA‐DS group (> − 0.74) were significantly longer than those in the nPSA‐DS group (≤ − 0.74) in the Kaplan–Meier survival curve (Figure 2).

The results from this study are consistent with those of previous studies showing that nadir PSA levels following PADT are a significant predictor of prognosis. In addition, we found that TTPN seems to be replaced by nPSA‐DS. We also observed a significant association between nPSA‐DS and TTPN, age and N stage during PADT (*p* < 0.05). The lower nPSA‐DS group (≤ −0.74) had a shorter TTPN, younger age, and higher N stage than the higher nPSA‐DS group (> − 0.74). However, the nPSA‐DS was determined primarily by TTPN after PADT.

Considering the effect of immortal time bias, nPSA, TTPN, nPSA‐DS, nPSA‐PDS, and nPSA‐LS, as time‐dependent covariates, were analyzed by multivariate Cox proportional hazards regression to confirm the significant factors for predicting PFS and OS. nPSA‐DS and nPSA were significant independent prognostic factors for PFS, and OS was supported by the analysis of nPSA‐DS and nPSA as time‐dependent covariates.

Although the combined analyses showed a potential synergistic effect of nadir PSA and TTPN on the outcome in previous studies, these factors have some pitfalls. In the combined analysis of TTPN and nadir PSA, Choueiri et al.^12^ and Huang et al.^30^ found that TTPN was independently associated with OS and PFS in patients with higher PSA nadir (≥0.2 ng/ml) but not in those with lower PSA nadir (<0.2 ng/ml). Sasaki et al.^14^ and Hong et al.^25^ showed that the relationship between nadir PSA and survival was limited only to patients with a shorter TTPN. Our data showed that the combined analysis of nPSA‐DS and nadir PSA can remedy those defects. However, the mechanism for the synergistic effect among these two variables needs further investigation.

Prostate cancer tissues may consist of a mixture of androgen‐sensitive cells and androgen‐resistant cells, and the ratio of androgen‐sensitive to androgen‐resistant cell composition varies among patients. The proportion of androgen‐sensitive cells in the cancer tissue was much higher than that of androgen‐resistant cells in those patients with a better prognosis, and the prostate volume decreased rapidly after ADT treatment.^31^, ^32^ Therefore, we hypothesized that the poor prognosis of the low nPSA‐DS group (≤ − 0.74) was associated with short TTPN, high iPSA, and high nPSA. This phenomenon may be caused by a lower percentage of androgen‐sensitive cells than androgen‐resistant cells and the rapid death of androgen‐sensitive cells during ADT treatment, resulting in shorter TTPN. The presence of a large number of androgen‐resistant cells resulted in higher iPSA and nPSA. In contrast, in the high nPSA‐DS group (> − 0.74), the proportion of androgen‐sensitive cells was higher than that of androgen‐independent cells. It may take more time for ADT treatment to kill a large number of androgen‐sensitive cells, resulting in a longer time for PSA to fall to the nadir.

Several limitations of this study should be recognized. First, it was a retrospective study from a single center. Therefore, the design of data collection during PADT was not defined prospectively or standardized. Second, our study had a small sample size, and this population may limit the generalization of our results. Third, the lack of information on the use of add‐on or second‐line treatment is a potential confounder that may have influenced our results. Nevertheless, we believe that this finding warrants further investigation.

In conclusion, our results indicated that the slope associated with the nadir PSA of nPSA‐DS was highly predictive of PFS and OS. Patients with nPSA‐DS > −0.74 have prolonged survival after PADT. The synergistic effect of nPSA‐DS and nadir PSA on prognosis has advantages over TTPN and nadir PSA in patients treated with PADT. Of course, our results need to be confirmed by further large‐scale prospective trials and validated externally.

## CONFLICT OF INTEREST

There are no conflict of interest to declare.

## AUTHOR CONTRIBUTIONS

Conceptualization: Wang Gongxian. Data acquisition: Zeng Zhenhao, Yi Ming, Zhang Hongtao, and Jiang Hao. Analysis and interpretation of data: Zeng Zhenhao, Zhang Cheng, and Cheng Xiaofeng. Statistical analysis: Zeng Zhenhao, He Wenrui Cheng Xiaofeng, and Jiang Hao. Manuscript writing: Zeng Zhenhao. Manuscript editing: Wang Gongxian and Zhou Xiaochen. All authors approved the submitted article.

## ETHICS STATEMENT

This study complies with the Helsinki Declaration. This study was reviewed by the Ethics Committee of the First Affiliated Hospital of Nanchang University and was judged as a “non‐applicable” research. Because this study was retrospective, does not produce any potential risks and benefits for the patients. The patients informed consent were waived.

## Data Availability

The data are available from the corresponding author
